# Organosilicon Compounds in Hot-Melt Adhesive Technologies

**DOI:** 10.3390/polym15183708

**Published:** 2023-09-08

**Authors:** Jakub Czakaj, Bogna Sztorch, Eliza Romanczuk-Ruszuk, Dariusz Brząkalski, Robert E. Przekop

**Affiliations:** 1 Faculty of Chemistry, Adam Mickiewicz University in Poznań, Uniwersytetu Poznańskiego 8, 61-614 Poznań, Poland; jakub.czakaj@amu.edu.pl; 2 Centre for Advanced Technologies, Adam Mickiewicz University in Poznań, Uniwersytetu Poznańskiego 10, 61-614 Poznań, Poland; bogna.sztorch@amu.edu.pl (B.S.); dariusz.brzakalski@amu.edu.pl (D.B.); 3 Almara Sp. Z o.o. Sp.k., 3/627 Mozarta, 02-736 Warsaw, Poland; 4 Institute of Biomedical Engineering, Faculty of Mechanical Engineering, Bialystok University of Technology, Wiejska 45C Street, 15-351 Bialystok, Poland; e.romanczuk@pb.edu.pl

**Keywords:** organosilicon, silanes, hot melt, HMA, adhesive

## Abstract

Hot-melt adhesives (HMAs) are thermoplastic materials that can bond various substrates by solidifying rapidly upon cooling from the molten state, and their modification with organosilicon compounds can result in crosslinking behavior, characteristic of gels. Organosilicon compounds are hybrid molecules that have both inorganic and organic components and can enhance the properties and performance of HMAs. The gel aspect of HMA with and without organosilicon modifiers can be considered in organosilicon-modified systems, the modifiers are often either sol–gel condensation products or their mechanism of action on the adherent surface can be considered of sol–gel type. The purpose of this manuscript is to present the current state of the art on the formulation, characterization, and application of HMAs and optimize their performance with organosilicon compounds for application in various industries such as automotive, construction, and photovoltaics. This review covers articles published within the period of 2018–2022. The article is divided into sections, in which information about hot-melt adhesives is described at the beginning. The following part of the presented review focuses on the composition of hot-melt adhesives, which takes into account the use of organosilicon compounds. The last part of this review outlines the future trends in hot-melt adhesives.

## 1. Introduction

### 1.1. Adhesive Bonding

**Figure 1:**
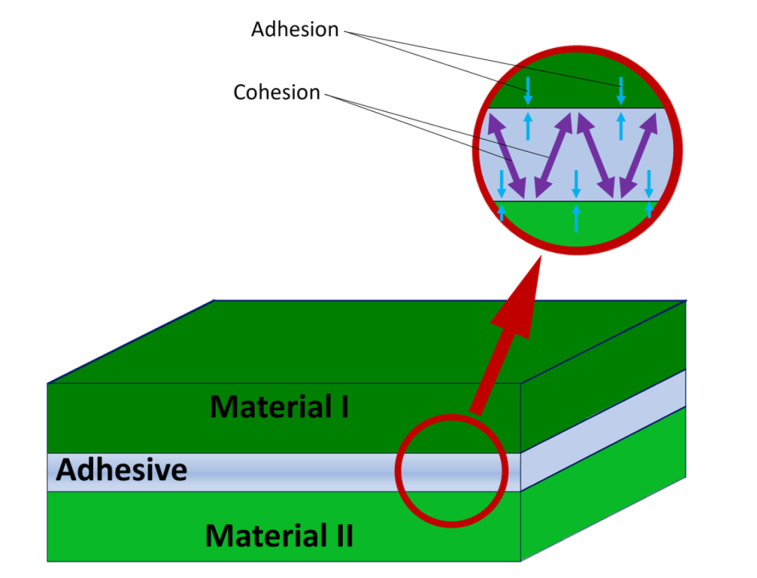
Scheme of adhesive bonding of two materials.

Adhesion refers to the process of forming a contact area between two materials that can sustain or transmit stress (Figure 1). It is the force that holds two solids together due to attractive surface forces. The classic work of Johnson, Kendall, and Roberts shows that surface energy and adhesion are related through the action of surface forces, such that the mechanical work needed to separate two solids in contact is equal to the loss of surface energy. The force of adhesion between elastic bodies depends on the geometry and energy of the contacting surfaces [1]. There are several interactions that contribute to the formation of an adhesive bond, including van der Waals attractive forces [1,2,3,4], mechanical interlocking [2,5,6,7,8,9,10,11], interdiffusion of polymer chains across the interface [12,13,14,15,16,17,18], chemical bonds at, or across the interface, and electrostatic interactions [11,19,20,21,22,23,24,25]. Currently, there is no comprehensive and quantitative theory that can establish a clear connection between the physical and chemical attributes of materials, and effectively explain their adhesion as well as the practical strength of an adhesive bond established with the said materials. However, the goal of adhesion science, which is the prediction of adhesive bond strength from the former interactions, can be reached by a proper combination of adhesion theories and a proper description of strain energy dissipation in the adhesive and adherend [26]. Two general functions generally govern the bond strength: The thermodynamic work of adhesion (*W*_0_), which is needed to separate the interface between the two materials in contact and is directly related to the surface energies of each material. The other is the fracture energy (*G*), which is related to the energy needed to create a unit surface of fracture. A general expression linking both values can be shown as:

$$G=W_{0}\left[1+\Phi \left(V,\;T,\ldots \right)\right]$$

where *Φ* is the amplifying factor, which depends on temperature, time, and any parameters that may alter the viscoelastic properties of the adhesive. *G* is typically a few orders of magnitude larger than *W*_0_ in the case of soft adhesives [27].

### 1.2. Hot-Melt Adhesive

Hot-melt adhesives (HMAs) have emerged as a significant area of interest in the field of adhesive technology, owing to their wide-ranging applications and unique properties. HMAs are thermoplastics that can effectively bond substrates of various materials by rapidly solidifying the applied material after cooling from the molten state [26,28]. Organosilicon compounds, on the other hand, are often products of sol–gel chemistry on their own, or their (or other organometallics) mechanism of action on the surface of the adherent can be considered of sol–gel type when they play a role of either surface primers or crosslinking agents with priming capabilities in such systems [29,30,31]. Sol–gel-formed, organosilicon-based hot-melt adhesives are known as well [32].

**Figure 2:**
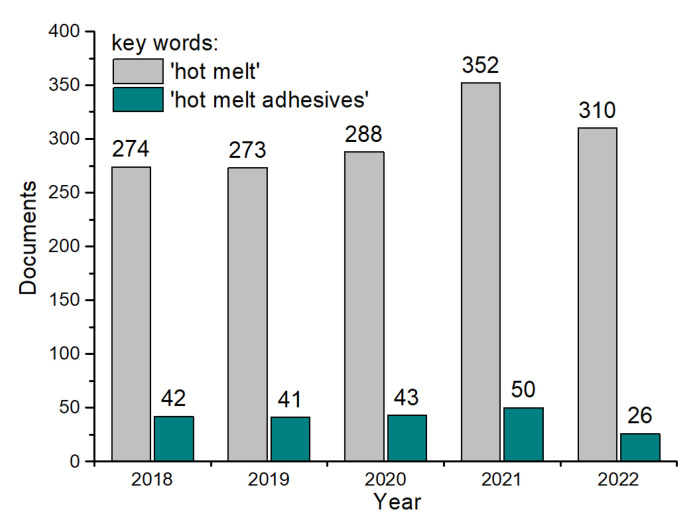
Scopus keywords: hot melt and hot-melt adhesives.

**Figure 3:**
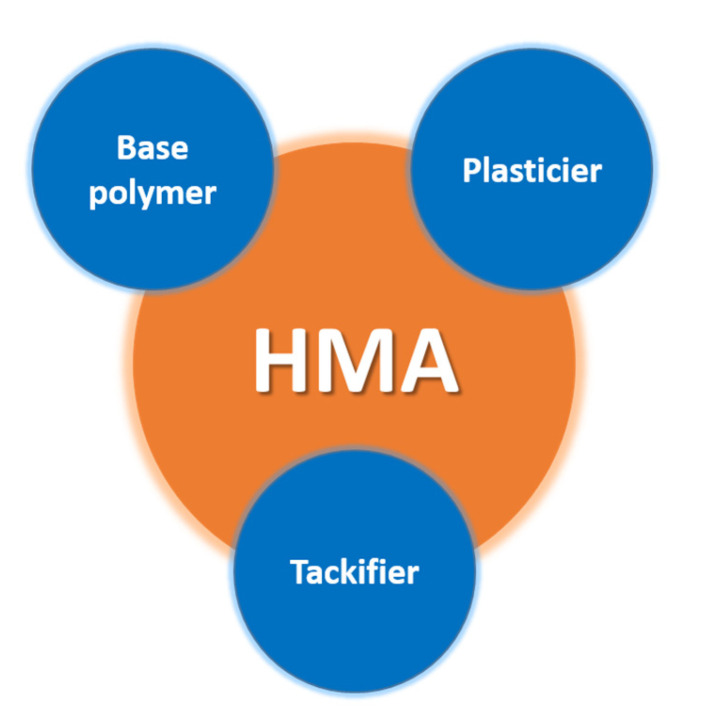
Main HMA ingredients.

Since the dawn of man, bitumen has been used in this role to stick rocks together [33,34]. In recent years, there has been a substantial increase in research and development of new formulations and applications for hot-melt adhesives, driven by the growing demand for high-performance, environmentally friendly, and cost-effective bonding solutions in various industries [35,36,37,38]. An analysis of the number of publications from the Scopus database in the years 2018–2022, in which the keywords: hot melt and hot-melt adhesives occur, shows that the largest number of articles on this issue was published in 2021 (Figure 2). At the same time, the same trend is visible for articles with the keyword: “hot melt”. In 2022, the number of articles with the keyword: hot-melt adhesives, was lower by half. However, during this time, there was a noticeable increase in publications on hot-melt modifiers and additives in the literature, as evidenced by the articles described in this manuscript. HMAs are solid at room temperature but become fluid and tacky when heated above their melting point. Upon cooling, they solidify rapidly, forming a strong bond between substrates [39]. This process is reversible, which means that hot-melt adhesives can be remelted and re-solidified multiple times, allowing for adjustments during the bonding process, or debonding, in the case of disassembly. The viscosity of the HMA system must remain low during the molten state to enable it to sufficiently wet the substrate surface. Therefore, to obtain sufficient bonding, the substrates must be joined during the open time of the HMA [40,41,42]. The fundamental components of HMAs include a polymer base, tackifiers, plasticizers, and other additives, such as stabilizers and fillers (Figure 3), which contribute to their unique characteristics and performance attributes.

HMAs have found widespread applications in various industries, such as packaging [35,43], automotive [44,45,46], electronics [47,48], woodworking [5], textiles [36], and solar cell and electronic device assembly [49,50]. In the packaging industry, for instance, HMAs are used for sealing boxes, labels, bookbinding, and flexible packaging materials, as well as bonding various components of consumer products. In the automotive sector, they serve as an essential material for assembling interior and exterior components, including upholstery, insulation, and trim. Additionally, HMAs have been employed in the electronics industry for potting and encapsulating sensitive components, as well as bonding heat sinks and other heat-sensitive parts.

As HMAs are formulated with rubbery polymers, they exhibit a wide deformability range, gap-filling capability, and fast bond formation limited by cooling time and barrier properties [47]. However, the thermoplastic properties of HMAs come at a cost. The heat and creep resistance are limited, a quick rise in viscosity during cooling hinders penetration into the substrate, and a rapid drop in toughness is expected with increased temperature [51].

## 2. Hot-Melt Adhesive Composition

### 2.1. Primary Resins and Ingredients of Hot Melts

The main constituents of hot-melt materials for glass sealants, corrosion protection, and water insulation consist of a base polymer, tackifier, and plasticizer. Among other usual materials are viscosity modifiers, extenders, UV stabilizers, antioxidants, and pigments. The composition and proportion of these ingredients significantly influence the performance and properties of the adhesive, such as bond strength, temperature resistance, open time, and adhesion to various substrates. The choice of base polymer for hot-melt formulation is often dictated by its end use and desired properties. The formulator must consider the desired processing method, working conditions, and price. In general, base polymers have a huge impact on viscosity and rheology, cohesive and adhesive strength, creep and tack, as well as processing and working temperature [39,40,51,52,53]. Common primary resins used in HMAs include ethylene–vinyl acetate (EVA) [38,43,45,49,50,54] polyolefins [54,55], styrenic block copolymers (SBC) [56,57], butyl rubber [58,59], polyamides [38,60], and reactive resins [48,61]. The modification of base polymer properties is obtained by the addition of tackifiers. The tackifiers are usually hydrocarbon resins, pure monomer resins, or rosin esters. They are typically selected to be miscible with the base polymer and their main role is the T_g_ modification. The aim of pure monomer resins with high T_g_ is mainly reinforcement of the formulation. Low T_g_ resins function typically as tackifiers by increasing the T_g_ of the elastomeric polymer (increasing the loss modulus simultaneously) and reducing its elastic modulus. In contrast, plasticizers function mainly as diluents, by reducing both the elastic and loss moduli, softening the formulation, and increasing creep.

The formulations based on SBCs utilize the phase separation of soft and hard blocks. The typical base polymers are of A-B-A triblock structure, with A being typically styrene end-block and B an elastomeric mid-block with T_g_ below room temperature. The end-block and mid-block should be thermodynamically immiscible, to provide a microphase-separated structure. The styrenic end-block, exhibiting T_g_ above the typical application temperature, provides physical crosslinks and prevents creep, whereas the soft elastomeric mid-block may be readily modified to meet the demands of the formulator. Owing to the different solubility parameters of both phases, each phase may be modified independently using additives with selective miscibility [62]. A notable example is styrene–ethylene–butene copolymer (SEBS), which exhibits excellent affinity for paraffinic oil, and no bleed. Hot-melt oil–gel-type adhesives for dermal applications, comprising more than 600 PHR (per hundred rubber/per hundred resin) may be successfully formulated [63]. Such adhesives serve usually as carriers for physiologically active agents. Notably, the liquid nature of the formulation improves the drug release rate. It has been shown by Gennari et al. [64], that, in particular, low molecular weight SEBS is the polymer worthy of consideration because of its favorable viscoelastic behavior. Not only are SEBS gels attractive in medical applications, but also the inherent tackiness and the elasticity of such gels allow a compromise between minimizing modulus (to allow the polymer to be stretched with ease) and maximizing interfacial adhesion strength at the laminated polymer–polymer interface. This allows potential applications in wearable electronics such as soft-actuated materials and transducers [65]. In combination with the deformability of liquid metals, this allowed the fabrication of a stretchable thermoplastic electric conductor [66].

**Figure 4:**
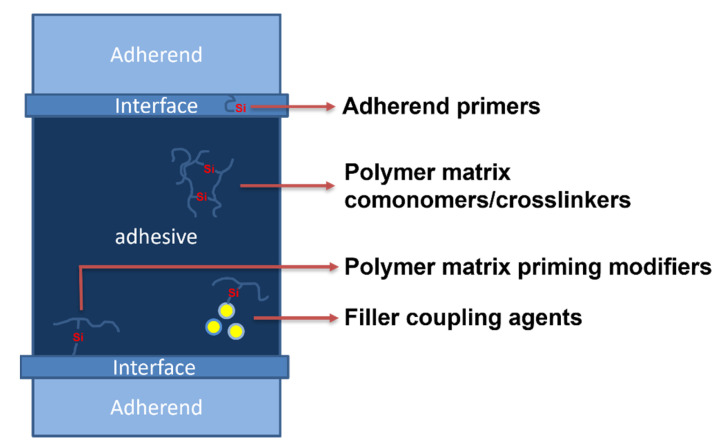
The application of organosilicon compounds within hot-melt adhesives and adhesive bonds.

Typical additives for butyl sealants are alkylphenol–formaldehyde resins (APFRs); gum rosin; hydrocarbon resins; low-molecular-weight polyisobutylene [67], paraffinic oil [68], C5 resin [69], high-molecular-weight polyisobutylene, silane-modified poly-alfa-olefin [70], butyl rubber, butadiene-styrene, and butadiene–nitrile rubbers, acrylic polymers, among other oligomers, polymers, and mixtures thereof, as well as natural and precipitated calcium carbonate silica [71], montmorillonites [71], sepiolite [72], carbon black [68], titanium dioxide [69], fumed silica, and halloysite [73]. Therefore, there is a large base of additives for HMA formulations, including organic and inorganic materials; however, usually, each one has a specific role, such as rheology or tackiness modifier, extender, pigment, etc. On the other hand, organosilicon compounds may serve multiple roles depending on their chemical structure, site, and method of application within either HMA itself or the adhesive bond formed with it (Figure 4). As mentioned earlier, they may be used to obtain silane-modified resins used for the bulk of HMA, such as silane-modified poly-alfa-olefin [70] (Figure 2A), or as various modifiers of fillers and adherents.

**Figure 5:**
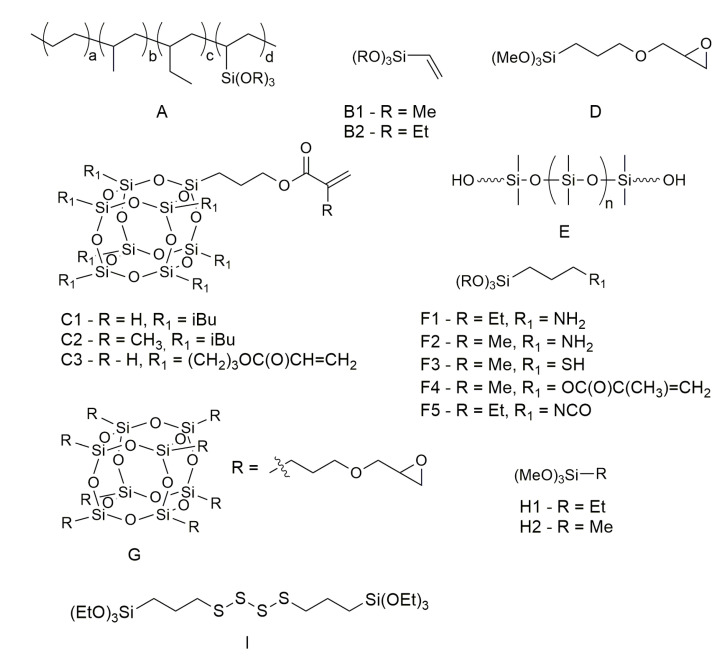
Structures of organosilicon agents discussed in the review.

Curable hot-melt sealants based on butyl rubber (BR) and containing thermoplastic resins like polyethylene (PE) and ethylene–vinyl acetate copolymer (EVA) exhibit better mechanical properties. It has been shown that an increase in the properties of sealants is due to the chemical interaction between EVA and vinyltrimethoxysilane (VTMOS, Figure 5B1) via transesterification reaction between the ethylene acetate monomers and alkoxysilane moiety [58].

### 2.2. Organosilicon Compounds as Co-Monomers of Polymer Matrix

The field of organosilicon polymer chemistry has achieved significant advancements over the past century, establishing a fundamental foundation for their utilization in materials science. Due to their unique inorganic–organic chemical composition, organosilicon polymers serve as a crucial link between inorganic and organic polymers, exhibiting a fascinating combination of properties [74,75,76]. Based on variations in their backbone structure, organosilicon polymers can be primarily classified into polysiloxanes (Si-O), polysilsesquioxanes (Si-O), polysilanes (Si-Si), polycarbosilanes (Si-C), and polysilazanes (Si-N) [77]. Kowalczyk et al. reported the synthesis and characterization of novel organic–inorganic hybrid copolymers based on acryloxypropyl-heptaisobutyl-POSS (A-POSS, Figure 5(C1)) and various (meth)acrylates, and their application in thermally curable structural self-adhesive tapes (SATs). The authors claim that the incorporation of A-POSS into the epoxyacrylate copolymers (EA-POSS) improves the self-adhesive, mechanical, and thermal properties of the SATs and the properties of the resulting aluminum–SAT–aluminum overlap joints. The results showed that the SATs with EA-POSS copolymers had higher adhesion, tack, and cohesion than the neat SAT-0, especially for lower A-POSS content (0.25–1 mol%). The best mechanical performance was observed for the joints with EA-POSS-0.5-based SAT, which had an increment range of 50–294% in shear strength compared to the EA-0-based joints. The results showed that the SATs with EA-POSS-type copolymers exhibited significantly higher values of adhesion, cohesion, and tack. The study concludes that A-POSS is able to improve the mechanical and thermal properties of SAT-based joints [78]. Ma et al. synthesized a series of POSS-containing linear and star multi-arm block copolymers (BCPs) with different architectures by the core-first atom transfer radical polymerization (ATRP) method and using methacryloxypropylheptaisobutylsilsesquioxane (Figure 5(C2)). They then crosslinked the BCPs by reacting the glycidyl groups in the poly(glycidyl methacrylate) (PGMA) block with trimethylamine to form three-dimensional networks (L/S-(PGMA-b-PMAPOSS)1,2,4,6) with various architectures. They found that the surface roughness and hydrophobicity increased with the number of arms of the BCPs, due to the higher aggregation and migration of PMAPOSS chains to the surface. The authors suggested that the surface properties could be tuned by adjusting the architectures of the BCPs. The authors measured the adhesive strength of the BCPs to glass substrates by a mechanics test system (MTS). They found that the adhesive strength increased with the number of arms of the BCPs, from 237 N for linear monobrachial BCPs to 431 N for star six-arm BCPs. They attributed this improvement to two factors: (1) the lower viscosity of the BCP solution with more arms, which enhanced its wetting and diffusion ability on glass surfaces; and (2) the higher density of branches of the BCPs with more arms, which increased their intermolecular forces and cohesion [79]. 

Hanifpour et al. [80] described a photo-crosslinkable adhesive by grafting methacrylic groups onto a co-oligomer of 1-decene and 9-decene-1-ol, which was prepared by using a Ti amine bis-phenolate catalyst. The adhesive was then blended with different weight fractions (0.2, 0.4, 0.7, and 1.0 wt.%) of a silsesquioxane referred to as methacrylate-functionalized silsesquioxane (meth-acryloxypropylsilsesquioxane, MA-POSS), although all the figures suggest that the additive was in fact acryloqypropylsilsesquioxane (Figure 5(C3)). The additive was of non-specified average molecular weight, and the obtained mixtures were cured by blue light irradiation. The results showed that the addition of MA-POSS increased the degree of monomer conversion, storage modulus, glass transition temperature, flexural modulus, flexural strength, microhardness, thermal stability, and adhesion properties of the nanocomposites. The authors attributed these improvements to the good dispersion and interfacial adhesion of MA-POSS in the adhesive matrix, as well as the increased crosslinking density and rigidity of the nanocomposites. However, they also observed that excessive MA-POSS content (1.0 wt.%) led to a reduction in mechanical and thermal properties due to the formation of aggregates and incomplete curing. The authors concluded that MA-POSS is an efficient adhesion promoter for olefin-based adhesives and can enhance their mechanical and thermal performance. They suggested that MA-POSS can be used for various industrial applications that require high-performance adhesives [80].

Bilgin et al. [81] reported the synthesis and characterization of 2-ethylhexyl acrylate (2-EHA)-based latexes via mini-emulsion polymerization for pressure-sensitive adhesive (PSA) applications. The authors investigated the effects of two types of silanes, vinyltrimethoxysilane (VTMOS) and 3-glycidyloxypropyltrimethoxysilane (GPTMOS, Figure 5(D)), on the adhesive performance of the latexes on polar and nonpolar surfaces. They also used n-dodecyl mercaptan (NDM) as both a cosurfactant and a chain transfer agent to control the particle size and molecular weight of the latexes. The authors found that mini-emulsion polymerization was an effective technique for incorporating silanes into 2-EHA-based copolymers without causing coagulation or instability. They also found that the type of silane had a considerable influence on the PSA properties. VTMOS, as a polymerizable silane, increased the shear strength of the latexes due to chemical crosslinking between chains, but decreased the peel adhesion and loop tack values on both polar and nonpolar surfaces. GPTMOS, as a non-polymerizable silane oligomer, increased the peel adhesion and loop tack values on both types of surfaces due to its epoxy functionality and polar interactions with substrates, but decreased the shear strength of the latexes. The work contributes to the understanding of the role of silanes in enhancing the adhesion and cohesion mechanisms of PSAs and offers an approach for tailoring the properties of PSAs according to specific requirements [81].

In the studies discussed above, organosilicon compounds were incorporated into the polymer matrix in different ways, such as through the synthesis of novel organic–inorganic hybrid copolymers, the creation of block copolymers (BCPs) with different architectures, and the grafting of methacrylic groups onto a co-oligomer. The use of organosilicon compounds as co-monomers resulted in polymers with improved properties, such as increased adhesion strength, thermal stability, and mechanical performance. In conclusion, organosilicon compounds have proven to be effective co-monomers in the synthesis of polymers, enhancing their properties and expanding their potential applications.

### 2.3. Organosilicon Compounds as a Polymer Matrix Additive

Park et al. (2020) [82] investigated the adhesion improvement of acrylic pressure-sensitive adhesive (PSA) to low-surface-energy substrates using silicone urethane dimethacrylates (SiUDMAs). By controlling the ratio of diisocyanate to carbinol-terminated PDMS of the terminating unit undisclosed by the supplier (Figure 5E), different types of SiUDMAs were obtained and introduced as additives to the acrylic PSA. The modifiers had urethane moieties imparting the miscibility of the oligomer with acrylic PSA and also have acrylate groups that can crosslink with UV irradiation. The results showed that SiUDMAs significantly improved the loop tack and peel strength of PSAs on low-surface-energy substrates without compromising their thermal stability. The improvement was more pronounced for SiUDMA2.0 (with IPDI:PDMS 2:0 ratio), which had a similar molecular weight to SiDMA (silicone dimethacrylate) but higher miscibility with acrylic PSA. UV irradiation decreased the loop tack and peel strength but increased the shear adhesion failure test (SAFT) of the modified PSAs due to the formation of a semi-interpenetrating polymer network (semi-IPN) structure by SiUDMA. The authors concluded that SiUDMAs are effective adhesion promoters for acrylic PSAs on low-surface-energy substrates and can overcome the limitations of SiDMA. They also suggested that SiUDMAs can be used to tailor the properties of PSAs by adjusting their molecular weight, viscosity, and surface energy. 

Wu et al. [83] reported on the effects of silane coupling agents on the properties of ethylene/vinyl acetate (EVA) composite hot-melt adhesive. The authors prepared a binary EVA resin blend with suitable viscosity and tensile shear strength as the base resin, and then added dicumyl peroxide (DCP) as the crosslinking agent and three types of silane coupling agents with different functional groups (KH550, KH560, and KH570, which are APTES, Figure 5(F1), GPTMOS, Figure 5(D), and MATMOS, Figure 5(F4), respectively) to improve the bonding performance of the adhesive. The optimal temperature and dosage of DCP for crosslinking EVA resin were 140 °C and 2 phr, respectively. After treatment under these conditions, the tensile shear strength of the adhesive increased from 0.247 MPa to 0.726 MPa when 5 phr KH570 was also added. The addition of the silane coupling agent reduced the degree of crosslinking of EVA resin by reacting with DCP preferentially, which resulted in a decrease in tensile strength, and elongation at the break and tensile modulus of the adhesive. However, KH570 had the lowest reactivity with DCP and improved the fluidity and wettability of the adhesive, as well as enhancing the polarity and bonding effect of the adhesive due to its methacryloxy functional group. The study found that when 2 phr of DCP and 5 phr of the KH570 silane coupling agent were added at the same time, the tensile shear strength of hot-melt adhesive increased from 0.247 MPa to 0.726 MPa. However, it was also found that an excessive silane coupling agent would significantly reduce the tensile strength and shear peel strength of the material. This may have contributed to the loss of entanglement [25]. Therefore, the addition of an appropriate amount of silicone coupling agent can improve the performance of EVA hot-melt adhesive. 

Yazıcı et al. (2021) [84] proposed a novel and environmentally friendly method to enhance the adhesion between natural rubber (NR) and textile cords, which are widely used in tire applications. The authors used acryloxypropyl-functional polyhedral oligomeric silsesquioxane (A-POSS), a reactive silsesquioxane species, as an additive in NR composites, and compared its performance with the conventional resorcinol formaldehyde latex (RFL) dipping system. A-POSS significantly increased the adhesion strength between NR composites and polyamide cords. The H-adhesion force between NR/A-POSS (8 phr) and virgin Nylon 6.6 cord was 123.0 N, while it was only 95.8 N for NR/RFL-coated Nylon 6.6 cord. The work of adhesion between NR/A-POSS (8 phr) and virgin Aramid was also higher than that of NR/virgin Aramid. The authors attributed these findings to the chemical reactions between A-POSS and sulfur during vulcanization, as well as the physical interactions between A-POSS and polyamide cords. They also observed a thin layer of NR/A-POSS on the surface of Nylon 6.6 cords by SEM images, indicating good interfacial adhesion.

The research paper by Murtazina et al. (2020) [85] presents a study of the effect of silane-terminated prepolymers (STP) based on oligotetraoxymethylene glycol (polyfurite) and oligooxypropylene glycol (laprol) on the properties of hot-melt sealants based on ethylene propylene diene rubber (EPDM). As a silane agent, 3-aminopropyltrimethoxysilane (Figure 5F2) was used. The results showed that the addition of STP increased the tensile strength and adhesion of the sealants to various substrates (duraluminum, steel, and glass), while reducing their elongation at break. The authors attributed this effect to the formation of a semi-interpenetrating network of cured STP in the uncured EPDM phase. They also found that the viscosity of the sealants decreased with increasing STP content, which indicated a temporary plasticization effect. The authors suggested that this could allow processing the sealants at lower temperatures (130 °C) than conventional hot-melt sealants (180–200 °C).

Lai et al. [86] investigated the thermal, mechanical, and shape memory behavior of physical blends of OBC (olefin block copolymer) and EVA (ethylene–vinyl acetate copolymer), with and without modification of one or both of the components via peroxide-initiated comonomer grafting reaction. The modification of EVA with vinyltriethoxysilane (VTEOS, Figure 5(B2)) improved the compatibility, tensile strength, and shape fixity ratio of the OBC/EVA blend. The OBC-g-MA/EVA-g-VTEOS blend, which had numerous interactions between maleic anhydride and silane, showed the highest storage modulus within the 60–80 °C range, thermal stability, and shape memory performance of all investigated blend systems. This modified blend could be reprocessed like a thermoplastic vulcanizate and, thus, could be considered a green shape memory blend in terms of environmental concerns. In summary, the use of silane as a modifying agent improved the adhesion and mechanical properties of OBC/EVA blends. 

### 2.4. Organosilicon Compounds as Filler Coupling Agents

Dognaci [87] investigated the use of glycidyl polyhedral oligomeric silsesquioxane (GPOSS, a glycidoxypropyl-functional silsesquioxane of non-specified molecular weight, Figure 5(G)) as an adhesion promoter to improve the adhesion between the polyester cord and rubber. The study found that the addition of GPOSS to poly(ethylene terephthalate) (PET) cord improved the adhesion of the cord to rubber when compared with other treated PET cords via H-adhesion and strip peel adhesion tests. The authors observed that GPOSS improved the adhesion of the cord to rubber significantly when compared with other treatments, especially at higher concentrations (0.5–1.0 wt.%). The authors attributed this improvement to the strong crosslinking and molecular reinforcement effects of GPOSS. In particular, the adhesion values were better than those obtained using commercially used epoxies, suggesting that GPOSS could be recommended as an adhesion promoter in the rubber industry. The study also found that GPOSS-coated PET yarns increased stiffness and did not change the tensile strength of the PET yarns. 

Yang et al. [11] investigated the interfacial adhesion between aramid fiber (AF) and rubber matrix by grafting mercapto hyperbranched polysiloxane (HPSi) onto the AFs via a novel in situ growth strategy. The HPSi was grafted via a combination of the formation of a polydopamine (PDA) precursor layer and the co-dehydration condensation between 3-aminopropyltrimethoxysilane (APTMOS) and 3-mercaptopropyltrimethoxysilane (MPTMOS, Figure 5(F3)). This modification strategy can increase the interfacial adhesion by up to approximately 96.5%, with the key factor being the covalent interaction between mercapto groups and double bonds. The study suggests that this surface modification strategy has the potential for application to other high-performance fibers and can expand the application range of fiber/rubber composites.

Ahmed and Mushtaq (2022) [88] studied the effects of silane-modified aluminum oxide (m-Al_2_O_3_) and ethylene–vinyl acetate-grafted maleic anhydride (EVA-g-MA)/m-Al_2_O_3_ hybrid fillers on the thermal stability and mechanical properties of ethylene–vinyl acetate copolymer (EVA)/ternary polyamide (tPA) composites. The authors used 3-aminopropyltriethoxysilane (APTES) as a coupling agent to modify the surface of Al_2_O_3_ particles and improve their dispersion and compatibility with the EVA/tPA matrix. They prepared the composites with different filler loadings (20–40 wt.%). Hybrid fillers modified with a silane coupling agent and EVA-g-MA were more effective in terms of improving both tensile and tear strength of such obtained materials. The tensile strength of the EVA/tPA/(m-Al_2_O_3_/EVA-g-MA) composite with a ratio of 49/21/30 wt.% increased by up to 66% compared to the neat EVA/tPA. The findings suggested that the silane modification and the EVA-g-MA compatibilizer approach for Al_2_O_3_ filler could be considered in future research work to develop reinforced composites of improved thermal stability. 

The work of Bi et al. (2019) [89] investigates the effects of four different silane coupling agents on the filler–filler and filler–rubber interactions and mechanical properties of the ethylene–vinyl acetate copolymer (EVM)/aluminum trihydrate (ATH) composites. The silane coupling agents were vinyltrimethoxysilane (VTMOS), vinyltriethoxysilane (VTEOS), ethyltrimethoxysilane (ETMOS, Figure 5H1), and aminopropyltrimethoxysilane (APTMOS). The dispersion and adhesion of ATH in the EVM matrix, and the tensile and abrasion properties of the obtained composites, are described. The main findings are that the addition of VTMOS, VTEOS, and APTMOS significantly reduces the Payne effect, which indicates the collapse of the filler network under shear and improves the tensile strength and abrasion resistance of the composites. The authors attribute this to the enhanced filler–rubber interaction mediated by the silane coupling agents, which have functional groups that can react with both ATH and EVM. On the other hand, ETMOS does not show any positive effect on the composites and even lowers the bound rubber content and mechanical properties. The authors suggest that ETMOS shields the ATH surface from wetting by EVM and does not participate in the peroxide curing reaction.

Jo et al. (2022) [90] reported on the development of a green and sustainable hot-melt adhesive (HMA) based on polyhydroxyalkanoate (PHA) and silanized cellulose nanofibers (SCNFs). The authors used PHA of a high chain length and high poly (4-hydroxybutyric acid) (P4HB) ratio as a biodegradable and flexible base polymer and modified the surface of cellulose nanofibers (CNFs) with tetraethyl orthosilicate (TEOS) and methyltrimethoxysilane (MTMOS, Figure 5H2) to enhance their hydrophobicity and dispersibility in PHA. The authors found that the double silanization of CNFs using TEOS and MTMOS increased their water contact angle from 18° to 177°, indicating a successful hydrophobization. They found that adding 10% SCNFs increased the tensile strength of PHA from 5.8 MPa to 7.2 MPa, while reducing its viscosity from 11,000 Pa·s to 9000 Pa·s. The authors attributed this to the thixotropic behavior of SCNFs, which enhanced the flowability and infiltration ability of PHA-SCNFs adhesive at low stress. The lap shear test showed that PHA-SCNFs adhesive had a comparable failure load to commercial HMA (around 1 kN), indicating a good adhesion performance. The authors concluded that PHA-SCNFs composite is a promising candidate for green and sustainable HMA applications, as it combines biodegradability, flexibility, processability, and adhesive strength. 

In conclusion, the exploration of organosilicon compounds as filler coupling agents in hot-melt adhesives has demonstrated significant potential across various applications. These compounds, characterized by their unique ability to form robust crosslinks and provide molecular reinforcement, have been shown to enhance the adhesion, mechanical properties, and thermal stability of diverse composite materials.

## 3. Materials Joined Using Hot Melts

### 3.1. Material Preparation 

#### 3.1.1. Physical Preparation (Sanding, Sandblasting, and Degreasing)

Sandblasting is a mechanical surface treatment method that can enhance adhesion strength and bonding performance. Sandblasting can modify the surface roughness and mechanical properties of the substrates by impacting them with abrasive particles of various hardness under different pressures and angles. The surface roughness can affect the surface free energy, wettability, and mechanical interlocking between the substrates and the adhesive. In a study by Li et al. [91], the authors used FM94 adhesive film, a modified epoxy film, to bond Al-Li alloy sheets that were subjected to sandblasting treatments using different parameters. The results showed that an increase in sandblasting pressure and abrasive size led to an increase in surface roughness, which contributed to the surface wettability and adhesion performance of the substrates. The bonding properties between Al-Li alloy and FM94 adhesive can be greatly improved by sandblasting treatment, and moderate surface roughness was found to yield better wettability and stronger shear strength. The maximum shear strength of the sandblasted sample was 31.5 MPa, while that of the sandpaper-treated reference sample was only 18.7 MPa.

#### 3.1.2. Chemical Preparation

**Figure 6:**
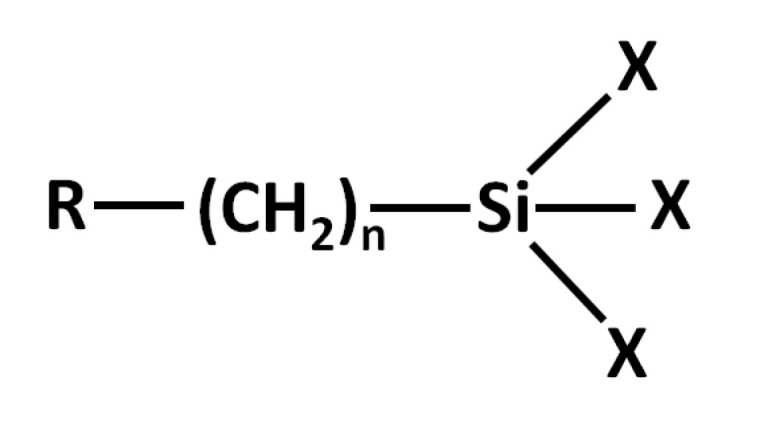
Structure of organofunctional silane.

One of the first areas of application of organosilicon compounds–organofunctional silanes (OFS), commonly referred to as silanes, was their use as coupling agents, primers, or adhesion promoters. The general formula (Figure 6) for organofunctional silanes is R(4-y)SiXy, where the R group is an organic group capable of binding or interacting with the surface of the substrate to be bonded to the polymer part. The X group is a halogen, an alkoxy group, or an acyloxy group.

The use of a silane coupling agent requires hydrolysis of the Si-X bond, or in rarer cases, a direct transesterification reaction with groups located on the bond surface. Due to the fact that the amount of the coupling agent in relation to the weight of the entire substrate is small, because it is important to create a monolayer (interphase, interlayer), diluted (0.25–2%) solutions of OFS in an organic solvent, often with a small amount of water and acid, are used to induce a hydrolysis reaction. Due to the high dilution, the condensation side reaction is limited. It should be noted that the mechanism of OFS binding to the surface is not exclusively covalent and its nature is much more complicated. Crosslinking bonds between silane molecules, weak interactions with the substrate surface, and similar interactions with the polymer mass have an equally significant and sometimes dominant effect on the overall interfacial phenomenon.

The use of organofunctional silanes as polymer–metal bonding compounds is interesting and has been studied in the past, as indicated by scientific articles from the 1990s [92,93]. Organofunctional silanes are often used as crosslinking agents for silicone elastomers that are cured with moisture. Therefore, they are used in many fields, including as seals in car engines, adhesives for electronic devices, anti-fouling coatings, and waterproofing seals in construction [92,94]. In addition, inorganic particle-modified organofunctional silanes are used to improve the strength of the interfacial bond between fiber and polymers in composite materials. Studies on modifying SiO_2_ with bis[3-(triethoxysilyl) propyl] tetrasulfide (TESPT, Figure 5I) silane and introducing it onto the surface of carbon fiber in a polymer–fiber composite have been described in the literature. The addition of silane improved the strength of the interfacial bond strength between polyamides and carbon fibers. It is worth noting that the addition of TESPT increased the shear strength between the layers [95]. Chen et al. showed that a gradient phase formed at the interface between the epoxy resin and carbon fiber, in which the surface graphene oxide was doped with APTES, provided good stress distribution at the interface when an external force was applied [95,96]. Hatefi et al. [97] investigated whether the GPTMOS layer would affect the adhesion of the epoxy coating to the aluminum substrate. One of the methods of protecting metals against corrosion is to apply a thin polymer layer on it. However, for this protection to be effective, it is important that this layer adheres to the metal. Various metal surface treatments have been used for this purpose, but they have not been sufficiently effective. There are reports that an additional coating between the polymer layer and the metal improves the adhesion of the polymer to the metal [98,99]. The additional coating, which uses organosilane compounds, acts as a primer, creating chemical bonds at the substrate–silane–polymer interfaces. Therefore, it can be used as a very effective adhesion promoter [97].

### 3.2. Materials for Hot-Melt Bonding

#### Glass

Glass is a popular adherend joined using hot melts. Hot-melt sealants are used in the manufacture of insulating glass windows and photovoltaic assemblies. They are also known as hot-flow sealants [98,99,100,101,102,103]. Numerous studies have demonstrated the detrimental impact of water on the mechanical characteristics of various metal oxide or glass-reinforced composites [104,105,106]. The diffusion and interaction of water at the filler–polymer interface cause the separation between the glass substrate (e.g., glass fiber) and polymer matrix [107]. To address these issues, coupling agents are employed to create a water-resistant connection between the polymer and glass, or any inorganic substrate or filler for that matter [108,109,110]. These coupling agents must have the capacity to react or engage with both the glass surface and the polymer to enhance the overall performance of the resulting reinforced composite materials [93,111,112,113]. Research has shown that incorporating even a small quantity of silane coupling agent into an inorganic filler can improve the performance of the resulting composite [106]. 

### 3.3. Organosilicon Coupling Agents for Glass Treatment

Planar glass surface, glass beads, glass fiber, or in general any surface with pendant silanol groups, may be modified using silane coupling agents. The overall structure of the molecules in question may be characterized as [R–SiX_3_], where the reactive substituent, denoted as X, may be a chlorine atom, a methoxy or ethoxy group, and labile and prone to hydrolysis and condensation, whereas the R moiety is comprised of functional groups such as aminoalkyl, epoxyalkyl, (meth)acryloxyalkyl, vinyl, or another hydrocarbon (mostly methyl, phenyl) group, and based on the structure of the substituent and the material of the adhesive behave as a reactive or inert substituent. The labile site located at the silicon site of the molecule engages with the GF (glass fiber) surface, whereas the other (reactive) site binds to the polymer matrix via either a chemical reaction or weak interactions [114]. Upon application, the silane coupling agent undergoes hydrolysis, producing silanols that are inherently unstable. These silanols, along with the hydroxyl groups on the surface of the fiber, merge through the removal of water molecules, giving rise to a covalently bonded siloxane network on the fiber surface. Due to its tendency for self-condensation, the resulting siloxane exhibits a low density and a proclivity towards hydrolysis and instability. When the fiber is subsequently integrated with the polymer matrix, the R substituent of the silane molecule engages with the reactive functional group of the polymer, thereby creating a robust network that serves to bridge the interface between the fiber and polymer [114,115]. Among the silane coupling agents, the following four are the most prevalent: 3-aminopropyl-triethoxysilane (APTES) [116,117], 3-glycidoxypropyltrimethoxysilane (GPTMOS) [118], 3-methacryloxypropyltrimethoxysilane (MATMOS) [115,119], and vinyltriethoxysilane (VTEOS) [120].

Polyolefin elastomer (POE) is a promising encapsulant material for photovoltaic (PV) modules due to its high transmittance, persistent bonding, and good creep resistance. However, POE has low adhesion to glass substrates, which may affect the reliability and durability of PV modules. To overcome this limitation, Park and Hwang (2022) [121] designed and synthesized a chemically modified hydrocarbon resin (m-HCR) using 3-methacryloxypropyltrimethoxysilane (MATMOS) as an adhesion promoter. They blended POE with different amounts of m-HCR and crosslinked them with dicumyl peroxide (DCP). The tensile properties of the uncrosslinked and crosslinked blends were also similar to those of neat POE, suggesting that the mechanical properties of POE were maintained with the addition of m-HCR. The peel strength between glass and encapsulant increased linearly with increasing m-HCR content, demonstrating that the silane moiety in m-HCR improved the adhesion to glass substrates. The PV modules utilizing the crosslinked POE/m-HCR blends as encapsulants showed a slight difference in electrical performance after manufacturing and damp-heat exposure for 1000 h. The overall conclusion was that POE/m-HCR blends with MATMOS as an adhesion promoter could be used as an adhesion-enhanced polyolefin encapsulant material for PV modules. However, care must be taken, as there are opposing results, suggesting that the use of silanes for PV encapsulation may result in their thermal degradation over time, and delamination of the modules [121,122,123], especially when the adhesive contains EVA.

#### 3.3.1. Metals

The adhesion of rubber to metal is an important technology for various applications such as tires, conveyor belts, and hydraulic tubes. However, the conventional method of using brass plating to enhance the adhesion between rubber and steel is costly and environmentally unfriendly. Therefore, alternative methods of improving the adhesion between rubber and steel are of great interest. Aluminum mesh, metalized film, and sheet aluminum are popular substrates in the building industry, working as a carrier or construction element for flashing tapes, roofing tape, or roof window collars [124,125,126]. Popular sound deadening and vibration damping plates (VDP) and tapes for the automotive industry are usually made of bitumen or butyl rubber-based adhesives [127]. A traditional VDP consists of a sandwich-like structure, where a viscoelastic rubber layer is placed between two steel plates, effectively absorbing vibrational energy [128,129]. Lately, aluminum has replaced steel as a means to decrease the weight and expense of VDPs [130,131]. Nonetheless, aluminum’s incompatibility with polymers compromises its ability to bond with the viscoelastic rubber in the VDP, leading to delamination issues during the pressing process in various applications [132]. Silane coupling agents, frequently utilized for surface modification, have emerged as an environmentally friendly adhesion promoter between metal and polymer [133,134,135,136,137,138].

#### 3.3.2. Organosilicon Coupling Agents for Metal Treatment

Li et al. [139] report a method to improve the adhesion strength and water resistance of stainless steel and ethylene acrylic acid/linear low-density polyethylene (EAA/LLDPE) blend film composites by using functionalized silane as a surface modifier. They found that the EAA/LLDPE (60:40) blend had the best balance of strength and toughness among the blends. They also treated stainless steel substrates with different volume ratios of 3-methacryloxypropyltrimethoxysilane (MATMOSand 3-aminopropyltrimethoxysilane (A-1110, APTMOS). They found that MATMOS and APTMOS were successfully grafted onto the stainless steel surface and formed chemical bonds with LLDPE and EAA, respectively. They then hot-pressed EAA/LLDPE films onto untreated or silane-treated stainless steel substrates and measured their peel strength before and after water resistance testing at 68 °C for 168 h. They found that silane treatment significantly enhanced the peel strength and water resistance of the composites, especially when a 3:7 MATMOS:APTMOS volume ratio was used. The peel strength after water resistance testing increased from 3.18 N/cm for untreated stainless steel to 9.37 N/cm for silane-treated stainless steel. They also observed rough and uniform voids on the peel surfaces of silane-treated composites by scanning electron microscopy (SEM), indicating strong interfacial interaction between the film and the substrate. They proposed a mechanism of energy dissipation involving mechanical energy dissipation and surface adhesion energy at the interface. 

Lee et al. [140] reported on improving the adhesion strength and damping performance of aluminum-based vibration damping plates (VDPs) with butyl rubber by applying 3-mercaptopropyltrimethoxysilane (MPTMOS) as a primer. The authors chose MPTMOS as the silane coupling agent because its thiol functional group can react with the double bonds within the butyl rubber polymer backbone via a thiolene reaction upon heating, forming covalent bonds between the two materials. The work describes the experimental methods and results of various surface analyses, T-peel strength tests, and central supporting vibration tests to evaluate the effects of MPTMOS treatment on the aluminum surface and the VDPs. The paper shows that MPTMOS treatment successfully formed a self-assembled monolayer on the aluminum surface, as confirmed by wetting contact angle, XPS, FT-IR, and AFM measurements. The optimal MPTMOS treatment time was found to be 7 min, which resulted in a 180% enhancement of peel strength compared to the untreated sample. MPTMOS treatment significantly improved the damping loss factor of the VDPs, achieving a sevenfold increase compared to the aluminum plate without MPTMOS. MPTMOS acts as a covalent bridging agent between aluminum and butyl rubber, where the silane group bonds to the aluminum surface by hydrolysis and condensation, forming Si-O-Al bonds, and the thiol group bonds to butyl rubber by a thiolene reaction, forming C-S bonds. This suggests that MPTMOS can be considered an eco-friendly and effective silane coupling agent for enhancing the adhesion and damping properties of aluminum VDPs with butyl rubber. 

Sang et al. (2017) [141] investigated the adhesion between carbon steel (CS) and natural rubber (NR) by using four different silane coupling agents with amino, thiol, glycidoxy, and isocyanate functional groups, that is, APTES, MPTMOS, GPTMOS, and ICPTEOS (Figure 5(F5)), respectively. They characterized the surface modification of CS by silane coupling agents and the adhesion strength between CS and NR by various techniques such as wetting contact angle measurements, peel testing, and local nanoscale thermal analysis. The authors found that APS was the most effective silane coupling agent for enhancing the adhesion between CS and NR, as it showed the highest peel strength and cohesive failure mode. They attributed this effect to the high surface coverage of APTES on CS, the low contact angle indicating high hydrophilicity, and the strong interaction between APS and NR through hydrogen bonding or covalent bonding. The other silane coupling agents also improved the adhesion between CS and NR compared to the blank CS, but to a lesser extent than APS. 

Picard et al. [18] investigated the bonding of two different grades of high-consistency silicone rubber (HCR) on aluminum studs using three primer formulations based on hydrolyzed and condensed vinyl-functional silanes. They characterized the properties of the primer films (thickness, roughness, and surface energy) and the primer/silicone elastomer interphases after the peeling test (hardness, fracture profile). They found that the primer formulations had different effects on the adhesion strength and failure mode of the HCR/metal composites, depending on the chemical composition and topology of the primer film and the mechanical properties of the HCR. The main conclusions of the study were that the primer film thickness varied from 0.6 to 5.6 micrometers depending on the formulation and coating method, as well as the surface energy, which ranged from 8 to 40 mN/m, with different polar and dispersive components depending on the alkoxysilane/silicon ratio. The adhesion strength of the HCR/metal composites, measured by a 90-degree peeling test, ranged from 1.2 to 9.8 N/mm depending on the primer formulation and HCR grade. The fracture profiles showed different percentages of adhesive, fine cohesive, and bulk cohesive failures depending on the primer film thickness, roughness, surface energy, and interpenetration with the HCR. The hardness decreased with increasing adhesive strength and cohesive failure percentage, indicating a softer interphase between the primer film and the HCR. The authors proposed that a balance between mechanical anchoring, interfacial interactions, interdiffusion, and chemical bonding was needed to achieve optimal adhesion.

#### 3.3.3. Plastics and Composites

A similar practical application to VDP, however, satisfying different requirements, is corrosion protection, where typically the adhesive is bound to the protected metal surface as well as a thermoplastic polymer shrink wrap. During the 1960s, polyethylene (PE) films were incorporated into the pliable butyl rubber compound to avert excessive stretching, significantly enhancing its stability [142]. This development was vital for its employment as corrosion prevention in the form of wrapping [143]. Inherently, butyl adhesives possess low oxygen permeability and do not facilitate diffusion or the transportation of water through their composition [144,145]. Similarly, polyethylene coatings show minimal water permeability, and water transportation does not occur with such chemical formulations [146]. By combining butyl elastomeric adhesives with a polyethylene layer, the passage of both water and oxygen through the coating is effectively impeded. As water and oxygen are the essential factors required for corrosion to transpire, their access to the steel substrate surface is essentially obstructed, thereby mitigating corrosion [147,148,149,150,151].

Tian and Guo (2021) reported a novel surface modification method for UHMWPE fibers using polyethylene wax grafted with methyl methacrylate (PEW-g-PMMA) alone or in combination with a silane coupling agent. They found that the PEW-g-PMMA coating introduced polar groups and increased the surface roughness of UHMWPE fibers, which enhanced the interfacial compatibility and bonding strength with epoxy resin. Moreover, the addition of a silane coupling agent further improved the interfacial adhesion by forming a three-dimensional network structure between UHMWPE fibers, PEW-g-PMMA, and epoxy resin. The authors used various characterization techniques such as the contact angle test and the single-fiber pull-out test to evaluate the effects of the coating temperature, grafting rate, and silane coupling agent concentration on the surface properties and interfacial performance of UHMWPE fibers. They proved that the optimal conditions for achieving the highest pull-out strength were 9 wt.% PEW-g-PMMA, 12 wt.% silane coupling agent, and 100 °C coating temperature. The authors concluded that the modification approach with PEW-g-PMMA alone or in combination with a silane coupling agent was effective in improving the interfacial adhesion performance of UHMWPE fiber without damaging its intrinsic properties [152].

The paper by Guo et al. (2019) investigated the effect of GPTMOS treatment on the adhesion of carbon fiber reinforced nylon 6 (CF/PA6) composite with aluminum alloy (AA6061-T4) using a modified Henkel 5089 epoxy adhesive with additional amine curing agents. Silane treatment improved the static strength of the adhesive-bonded CF/PA6-CF/PA6 and CF/PA6-AA6061 by 23% and 21%, respectively, compared to the bare adherends. Silane treatment formed Si-O-Si and Si-N covalent bonds on the surface of CF/PA6 and Si-O-Si and Al-O-Si covalent bonds on the surface of AA6061-T4, which enhanced the bond adhesion between the silane coating, adhesive, and adherends. Abrasion pretreatment prior to GPTMOS treatment further increased the static strength of the adhesive-bonded CF/PA6-CF/PA6 by 20% and facilitated the formation of Si-N functional groups in the silane coating by removing contaminants and weak layers on the surface of CF/PA6 [153].

#### 3.3.4. Organosilicon Treatment of Polymers

Woong et al. [154] demonstrated that by introducing epoxy or amine groups on the polycarbonate (PC) surface, they could increase the chemical affinity and crosslinking ability of PC with epoxy-based adhesives. The SEM images showed that the PC surfaces functionalized with GPTMOS and APTMOS had irregular bulging polymeric structures, indicating the presence of silane coupling agents on the surface. The shear strength measurements showed that compared to neat PC, which had a shear strength of ~363 N, PC-GPTMOS and PC-APTMOS had shear strengths of 611 N (~168% increase) and 594 N (~163% increase), respectively. In contrast, ultraviolet ozone-treated PC and O_2_ plasma-treated PC showed negligible increases in shear strength. 

## 4. Future Trends in Hot-Melt Adhesives

Based on the review that was carried out in this manuscript, it was noted that an important future issue relates to understanding the correlations between the structure, composition, and properties of HMAs. In addition, it was noticed that the interaction between the components in the HMA system may be related to the functionality and processing conditions, further affecting properties such as setting speed and mechanical properties (toughness, strength). One of the important aspects in the development of new HMAs is to increase the binding rate. To achieve this, new additives based on different polymeric compounds are constantly being developed.

## 5. Conclusions

The topic of improving the adhesion and performance of hot-melt adhesives for various applications is still challenging and requires further research to develop novel formulations and methods that can meet the increasing demands for high-performance, environmentally friendly, and cost-effective bonding solutions. In all the works reviewed, the authors demonstrate the effectiveness of organosilicon compounds as co-monomers, additives, or coupling agents for enhancing the properties and compatibility of hot-melt adhesives with different substrates, such as glass, metals, plastics, and composites. The incorporation of organosilicon compounds into the polymer matrix or the surface modification of fillers or adherends with silane coupling agents can improve the adhesion strength, thermal stability, mechanical performance, and damping behavior of hot-melt adhesives. A deep understanding of the chemistry and physics of the interactions between organosilicon compounds and other materials is essential for optimizing the design and processing of hot-melt adhesives. Additionally, proper selection of materials in terms of their relative properties and environmental impact cannot be overlooked in order to ensure the reliability and sustainability of HMAs. Therefore, it is important for researchers and practitioners in the field of hot-melt adhesives to keep abreast of the latest developments and innovations in organosilicon chemistry and technology. Moreover, it should be noted that the diversity and complexity of hot-melt adhesive systems pose significant challenges for the standardization and comparison of results among different studies. Hence, there is a need for more systematic and comprehensive investigations on the effects of organosilicon compounds on hot-melt adhesives.

## Data Availability

No new data were created, or where data is unavailable due to privacy or ethical restrictions.

## Abbreviations

2-EHA	2-ethylhexyl acrylate
AF	aramid fibers
AFM	Atomic Force Microscopy
APFR	alkylphenol–formaldehyde resin
A-POSS	acryloxypropyl-functional silsesquioxane
APTES	3-aminopropyltrimethoxysilane
APTMOS	aminopropyltrimethoxysilane
ATH	aluminum trihydrate
ATRP	atom transfer radical polymerization
BCP	block copolymer
BR	butyl rubber
CF	carbon fiber
CNFs-	cellulose nanofibers
CS	carbon steel
DCP	dicumyl peroxide
EA	epoxyacrylate copolymer
EAA	ethylene–acrylic acid copolymer
EPDM	ethylene propylene diene monomers rubber
ETMOS	ethyltrimethoxysilane
EVA	ethylene–vinyl acetate
EVA-g-MA	ethylene–vinyl acetate grafted with maleic anhydride
EVA-g-VTEOS	ethylene–vinyl acetate grafted with vinyltriethoxysilane
EVM	ethylene–vinyl acetate copolymer rubber (an abbreviation used by some manufacturers)
FT-IR	Fourier-Transform Infrared
GPOSS	glycidoxypropyl-functional silsesquioxane
GPTMOS	3-glycidyloxypropyltrimethoxysilane
HMA	hot-melt adhesives
ICPTEOS	3-cyanatopropyltriethoxysilane
IPN	inter-penetrating network
LLDPE	linear low-density polyethylene
m-HCR	chemically modified hydrocarbon resin
MA-POSS	methacryloxypropylsilsesquioxane
MATMOS	methacryloxypropyltrimethoxysilane
MPTMOS	3-mercaptopropyltrimethoxysilane
MTMOS	methyltrimethoxysilane
MTS	mechanics test system
NDM	N-dodecyl mercaptan
NR	natural rubber
OBC	olefin block copolymer
OBC-g-MA	olefin block copolymer grafted with maleic anhydride
P4HB	poly (4-hydroxybutyric acid)
PC	polycarbonate
PDA	polydopamine
PE	polyethylene
PET	poly(ethylene terephthalate)
PEW-g-PMMA	polyethylene wax grafted with methyl methacrylate
PGMA	poly(glycidyl methacrylate)
PHA	polyhydroxyalkanoate
POE	polyolefin elastomer
POSS	polyhedral oligomeric silsesquioxane (registered trademark of Hybrid Plastics)
PSA	pressure-sensitive adhesive
PV	photovoltaics
RFL	resorcinol formaldehyde latex
SAFT	shear adhesion failure test
SAT	self-adhesive tapes
SCCNFs	silanized cellulose nanofibers
STP	silane terminated prepolymers
TEOS	Tetraethoxysilane
tPA	ternary polyamide
UHMWPE	ultrahigh-molecular-weight polyethylene
UV	ultraviolet
VDPs	vibration damping plates
VTEOS	vinyltriethoxysilane
VTMOS	vinyltrimethoxysilane
XPS	X-ray Photoelectron Spectroscopy

## References

[1] Johnson K.L., Kendall K., Roberts A.D. (1971). Surface energy and the contact of elastic solids. Proc. R. Soc. A Math. Phys. Eng. Sci..

[2] Liu W., Li H., Zhu H., Xu P. (2020). The Interfacial Adhesion Performance and Mechanism of a Modified Asphalt–Steel Slag Aggregate. Materials.

[3] Owens D.K. (1970). Some thermodynamic aspects of polymer adhesion. J. Appl. Polym. Sci..

[4] Wang Y., Guo Z., Liu W. (2021). Adhesion behaviors on four special wettable surfaces: Natural sources, mechanisms, fabrications and applications. Soft Matter.

[5] Smith M.J., Dai H., Ramani K. (2022). Wood–thermoplastic adhesive interface—Method of characterization and results. Int. J. Adhes..

[6] Qiu B., Sun T., Li M., Chen Y., Zhou S., Liang M., Zou H. (2020). High micromechanical interlocking graphene ox-ide/carboxymethyl cellulose composite architectures for enhancing the interface adhesion between carbon fiber and epoxy. Compos. A Appl. Sci..

[7] Lee J., Park E., Fujisawa A., Lee H. (2021). Diatom Silica/Polysaccharide Elastomeric Hydrogels: Adhesion and Interlocking Synergy. ACS Appl. Mater. Interfaces.

[8] Seong M., Park H.-H., Hwang I., Jeong H.E. (2019). Strong and Reversible Adhesion of Interlocked 3D-Microarchitectures. Coatings.

[9] Zhu M., Zhang F., Chen X. (2020). Bioinspired Mechanically Interlocking Structures. Small Struct..

[10] Hamilton A., Xu Y., Kartal M.E., Gadegaard N., Mulvihill D.M. (2021). Enhancing strength and toughness of adhesive joints via micro-structured mechanical interlocking. Int. J. Adhes. Adhes..

[11] Yang X., Tu Q., Shen X., Jiang C., Pan M., Zhu P., Li Y., Hu C., Zhang Q. (2020). Study on interfacial adhesion of the aramid fibers/rubber matrix by grafting mercapto hyperbranched polysiloxane. Polym. Test..

[12] Voiutskii S.S. (1963). Autohesion and Adhesion of High Polymers.

[13] Daelemans L., Paepegem W.V., Clerck K.D. (2018). Excellent Nanofiber Adhesion for Hybrid Polymer Materials with High Toughness Based on Matrix Interdiffusion During Chemical Conversion. Adv. Funct. Mater..

[14] Rathner R., Leimhofer C., Roland W., Hammer A., Löw-Baselli B., Steinbichler G., Hild S. (2022). Improving Layer Adhesion of Co-Extruded Polymer Sheets by Inducing Interfacial Flow Instabilities. Polymers.

[15] Shiraki Y., Saito M., Yamada N.L., Ito K., Yokoyama H. (2023). Adhesion to Untreated Polyethylene and Polypropylene by Needle-like Polyolefin Crystals. Macromolecules.

[16] Padhye N., Vallabh A. (2021). Deformation-induced bonding of polymer films below the glass transition temperature. J. Appl. Polym. Sci..

[17] Zaikov G.E., Yakh’yaeva K.S., Magomedov G.M., Kozlov G.V. (2020). Polymer Adhesion Model. J. Nat. Sci. Sustain. Technol..

[18] Picard L., Phalip P., Fleury E., Ganachaud F. (2015). Bonding of silicone rubbers on metal (2) physical chemistry of adhesion. Prog. Org. Coat..

[19] Gutowski W., Lee L. (1991). Thermodynamics of Adhesion. Fundamentals of Adhesion.

[20] Comyn J. (2021). Adhesion Science.

[21] Cui C., Liu W. (2021). Recent advances in wet adhesives: Adhesion mechanism, design principle and applications. Prog. Polym. Sci..

[22] Xie L., Gong L., Zhang J., Han L., Xiang L., Chen J., Liu J., Yan B., Zeng H. (2021). A wet adhesion strategy via synergistic cation–π and hydrogen bonding interactions of antifouling zwitterions and mussel-inspired binding moieties. J. Mater. Chem. A.

[23] Xu Y.Z., Liu J. (2021). Multiple H-bonding chain extender-based ultrastiff thermoplastic polyurethanes with autonomous self-healability, solvent-free adhesiveness, and AIE fluorescence. Adv. Funct. Mater..

[24] Zhang Q., Li T., Duan A., Dong S., Zhao W., Stang P.J. (2019). Formation of a supramolecular polymeric adhesive via water-participant hydrogen bond formation. J. Am. Chem. Soc..

[25] Wool R.P., Bunker S.P. (2007). Polymer-Solid Interface Connectivity and Adhesion: Design of a Bio-Based Pressure Sensitive Adhesive. J. Adhes..

[26] Malysheva G.V., Bodrykh N.V. (2011). Hot-melt adhesives. Polym. Sci. -D.

[27] Benedek I., Feldstein M.M. (2008). Fundamentals of Pressure Sensitivity.

[28] Li Z., Zhu W., Li R., Zeng Z., Han T., Ma Z. (2012). Method for preparing polyester hot melt gel special for tin can seal, China. CN Patent.

[29] Li J., Bai H., Feng Z. (2023). Advances in the Modification of Silane-Based Sol-Gel Coating to Improve the Corrosion Resistance of Magnesium Alloys. Molecules.

[30] Yan W., Chen B., Mahurin S.M., Hagaman E.W., Dai S., Overbury S.H. (2004). Surface Sol−Gel Modification of Mesoporous Silica Materials with TiO_2_ for the Assembly of Ultrasmall Gold Nanoparticles. J. Phys. Chem. B.

[31] Chen X., Wilson G.S. (2004). Electrochemical and Spectroscopic Characterization of Surface Sol−Gel Processes. Langmuir.

[32] Schmidt H., Scholze H., Tünker G. (1986). Hot melt adhesives for glass containers by the sol-gel process. J. Non-Cryst. Solids.

[33] Feldstein M.M., Siegel R.A. (2012). Molecular and nanoscale factors governing pressure-sensitive adhesion strength of viscoelastic polymers. J. Polym. Sci. B Polym. Phys..

[34] (2004). Molecular Adhesion and Its Applications.

[35] Fay P.A. (2021). A History of Adhesive Bonding. Adhesive Bonding.

[36] Gadhave R.V.I., Gadhave C.R. (2022). Adhesives for the Paper Packaging Industry: An Overview. Open J. Polym. Chem..

[37] Gharde S., Sharma G., Kandasubramanian B. (2021). Hot-Melt Adhesives: Fundamentals, Formulations, and Applications: A Critical Review. Prog. Adhes. Adhes..

[38] Orgilés-Calpena E., Arán-Aís F., Torró-Palau A.M., Sánchez M.A.M. (2017). Adhesives in the footwear industry: A critical review. Rev. Adhes.

[39] Vineeth S.K., Gadhave R.V. (2020). Sustainable raw materials in hot melt adhesives: A review. Open J. Polym. Chem..

[40] Mittal K.L., Pizzi A. (2020). Handbook of Sealant Technology.

[41] Petrie E.M. (2021). Handbook of Adhesives and Sealants.

[42] Yarusso D.J. (2002). Effect of rheology on PSA performance. Adhesion Science and Engineering.

[43] Pocius A.V., Pocius A.V. (2021). 11—Thermoplastic, Pseudothermoplastic, and Other Adhesives. Adhesion and Adhesives Technology.

[44] Moyano M.A., París R., Martín-Martínez J.M. (2019). Viscoelastic and adhesion properties of hot-melts made with blends of ethylene-co-n-butyl acrylate (EBA) and ethylene-co-vinyl acetate (EVA) copolymers. Int. J. Adhes. Adhes..

[45] Ciardiello R., Belingardi G., Martorana B., Brunella V. (2019). Physical and mechanical properties of a reversible adhesive for automotive applications. Int. J. Adhes. Adhes..

[46] Sharma S., Sharma B., Manral A., Bajpai P.K., Jain P. (2020). Biopolymers in the automotive and adhesive industries. Biopolymers and Their Industrial Applications.

[47] Konoplin A.Y., Pushkarev A.V., Shakurov A.V., Baurova N.I. (2021). Evaluation of Frost Resistance of Organosilicon Sealant at Ultralow Temperatures. Polym. Sci. Ser. D.

[48] Adkinson D.K. (2016). Hot Melt Adhesives with Butyl Ionomer. European Patent Office, Patent.

[49] Wang S., Liu Z., Zhang L., Guo Y., Song J., Lou J., Guan Q., He C., You Z. (2019). Strong, detachable, and self-healing dynamic crosslinked hot melt polyurethane adhesive. Mater. Chem. Front..

[50] Beaucarne G., Zelba M., Jadot E., Curon J., Gubbels F., Hayez V., Arenas B.S., Chambard G., Karoblis R. Low Temperature Solar Cell Encapsulation with Novel Silicone Elastomer for Building Integrated Pv. Proceedings of the 8th World Conference on Photo-Voltaic Energy Conversion.

[51] Ramírez E., Betancur R., Montoya J.F., Velilla E., Ramírez D., Jaramillo F. (2022). Encapsulation against Extrinsic Degradation Factors and Stability Testing of Perovskite Solar Cells. Recent Advances in Multifunctional Perovskite Materials.

[52] da Silva L.F.M., Öchsner A., Adams R.D. (2018). Handbook of Adhesion Technology.

[53] Pizzi A., Mittal K.L. (2017). Handbook of Adhesive Technology.

[54] Cherkashina A., Rassokha O., Mazhuga O. (2021). Melting Adhesives with High Adhesion. Ph.D Thesis.

[55] Robertson D., van Reenen A., Duveskog H., Brady F. (2021). A comparative study of the application-based properties of hot melt adhesives (HMAs) formulated with different waxes. Int. J. Adhes. Adhes..

[56] Varga L.J., Bárány T. (2021). Development of recyclable, lightweight polypropylene-based single polymer composites with amorphous poly-alpha-olefin matrices. Compos. Sci. Technol..

[57] Anderson J.J. (2021). Styrenic Block Reinforcing Additives in Pressure Sensitive Adhesives.

[58] Maji P., Naskar K. (2022). Styrenic block copolymer-based thermoplastic elastomers in smart applications: Advances in synthesis, microstructure, and structure-property relationships—A review. J. Appl. Polym. Sci..

[59] Lisanevich M.S., Galimzyanova R.Y., Rusanova S.N., Khakimullin Y.N., Stoyanov O.V. (2018). Hot-Melt Sealants of Curable Type Based on Butyl Rubber and Ethylene–Vinyl Acetate Copolymer. Polym. Sci. Ser. D.

[60] Behera P.K., Kumar A., Mohanty S., Gupta V.K. (2022). Overview on Post-Polymerization Functionalization of Butyl Rubber and Properties. Ind. Eng. Chem. Res..

[61] Pourali M., Peterson A.M. (2022). A tale of two polyamides: Comparing the crystallization kinetics of a hot-melt adhesive and a PA 6/66 copolymer. Thermochim. Acta.

[62] Hu Y., Paul C.W., Benedek M.M. (2009). Block Copolymer-Based Hot-Melt Pressure-Sensitive Adhesives. Feldstein Technology of Pressure-Sensitive Adhesives and Products.

[63] Paul C.W. (2002). Hot Melt Adhesives for Dermal Application. U.S. Patent.

[64] Gennari C.G.M., Quaroni G.M.G., Creton C., Minghetti P., Cilurzo F. (2020). SEBS block copolymers as novel materials to design transdermal patches. Int. J. Pharm..

[65] Liu Z., Liu Y.D., Shi Q., Liang Y. (2021). Electroactive dielectric polymer gels as new-generation soft actuators: A review. J. Mater. Sci..

[66] Mineart K.P., Lin Y., Desai S.C., Krishnan A.S., Spontak R.J., Dickey M.D. (2013). Ultrastretchable, cyclable and recyclable 1- and 2-dimensional conductors based on physically cross-linked thermoplastic elastomer gels. Soft Matter.

[67] Wu M., Liu Y., Du P., Wang X., Yang B. (2020). Polyurethane hot melt adhesive based on Diels-Alder reaction. Int. J. Adhes. Adhes..

[68] Galimzyanova R.Y., Lisanevich M.S., Khakimullin Y.N. (2021). The Effect of Adhesive Additives on the Properties of Uncured Sealants Based on Butyl Rubber. Polym. Sci. Ser. D.

[69] Galimzyanova R.Y., Lisanevich M.S., Khakimullin Y.N. (2021). The Effect of Carbon Black on Characteristics of Nonhardening Sealants Based on Butyl Rubber. Polym. Sci. Ser. D.

[70] Czakaj J. (2018). Otrzymywanie częściowo usieciowanych mieszanek uszczelniających za pomocą wytłaczarki dwuślimakowej współbieżnej. Przemysł Chem..

[71] Czakaj J. (2018). Kompozytowe termoplastyczne uszczelniacze butylowe o zwiększonej odporności na wysoką temperature. Przemysł Chem..

[72] Brantseva T.V., Antonov S.V., Kostyuk A.V., Ignatenko V.Y., Smirnova N.M., Ilyin S.O. PIB Pressure-sensitive Adhesives with Dispersed Nanofillers: Addressing the Cold Flow Problem. Proceedings of the Conference: 11th European Adhesion Conference (EURADH 2016).

[73] Kumar K.D., Tsou A.H., Bhowmick A.K. (2010). Unique Tackification Behavior of Needle-like Sepiolite Nanoclay in Brominated Isobutylene-*co*-*p*-methylstyrene (BIMS) Rubber. Macromolecules.

[74] Kostyuk A., Ignatenko V., Smirnova N., Brantseva T., Ilyin S., Antonov S. (2014). Rheology and adhesive properties of filled PIB-based pressure-sensitive adhesives. I. Rheology and shear resistance. J. Adhes. Sci. Technol..

[75] Jones R.G., Ando W., Chojnowski J. (2013). Silicon-Containing Polymers: The Science and Technology of Their Synthesis and Applications.

[76] Muzafarov A.M. (2010). Silicon Polymers.

[77] Shi H., Yang J., Li Z., He C. (2020). Introduction of Organosilicon Materials. Silicon Containing Hybrid Copolymers.

[78] Kowalczyk A., Kowalczyk K., Gziut K. (2019). Synthesis of Monoacryloxypropyl-POSS-based Hybrid Epoxyacrylate Copolymers and Their Application in Thermally Curable Structural Self-Adhesive Tapes. Polymers.

[79] Ma Y., Wu H., Shen Y. (2022). Dual-functional linear and star POSS-containing organic-inorganic hybrid block copolymers: Synthesis, self-assembly, and film property. J. Mater. Sci..

[80] Hanifpour A., Bahri-Laleh N., Nekoomanesh-Haghighi M. (2020). Methacrylate-functionalized POSS as an efficient adhesion promoter in olefin-based adhesives. Polym. Eng. Sci..

[81] Bilgin E.T., Dülgar C.A., Serhatlı İ.E. (2019). Incorporation of vinyl silane and epoxy silane oligomer into 2-EHA-based poly-acrylate latexes via mini-emulsion polymerization and investigation of pressure-sensitive adhesive properties on polar and nonpolar surfaces. Polym. Bull..

[82] Park H.-W., Seo H.-S., Lee J.-H., Shin S. (2020). Adhesion improvement of the acrylic pressure-sensitive adhesive to low-surface-energy substrates using silicone urethane dimethacrylates. Eur. Polym. J..

[83] Wu Z., Shangguan Y., Zhang C., Zheng Q. (2021). Effects of Crosslinking and Silicone Coupling Agent on Properties of EVA Composite Hot Melt Adhesive. Polymers.

[84] Yazıcı N., Dursun S., Yarıcı T., Kılıç B., Arıcan M.O., Mert O., Karaağaç B., Özkoç G., Kodal M. (2021). The outstanding interfacial adhesion between acrylo-POSS/natural rubber composites and polyamide-based cords: An environmentally friendly alternative to resorcinol-formaldehyde latex coating. Polymer.

[85] Murtazina I., Akhmedgoraeva L., Galimzyanova A.R., Khakimullin R.Y. (2020). Construction Sealants Based on EPDM Modified with Silane-Terminated Urethane Prepolymers. IOP Conf. Ser. Mater. Sci. Eng..

[86] SLai M., Li C.-H., Kao H.-C., Liu L.-C. (2019). Shape Memory Properties of Melt-Blended Olefin Block Copolymer (OBC)/Ethylene-Vinyl Acetate Blends. J. Macromol. Sci. B.

[87] Doganci E. (2021). Improving adhesion between polyester cord and rubber by using glycidyl-POSS. J. Appl. Polym. Sci..

[88] Ahmed J., Mushtaq S. (2022). Effects of silane-modified Al_2_O_3_ and its hybrid filler on thermal stability and mechanical properties of ethylene–vinyl acetate copolymer/polyamide composites. Iran. Polym. J..

[89] Bi W., Hoch M., Yu G., Goegelein C., Kirchhoff J., Zhao S. (2019). Investigation of Silane Coupling Agents on the Filler-Filler and Filler-Rubber Interaction and Mechanical Properties of EVM/ATH Composites. IOP Conf. Ser. Mater. Sci. Eng..

[90] Jo J., Jeong S.-Y., Lee J., Park C., Koo B. (2022). Green and Sustainable Hot Melt Adhesive (HMA) Based on Polyhydroxyal-kanoate (PHA) and Silanized Cellulose Nanofibers (SCNFs). Polymers.

[91] Li J., Li Y., Huang M., Xiang Y., Liao Y. (2018). Improvement of aluminum lithium alloy adhesion performance based on sand-blasting techniques. Int. J. Adhes..

[92] Baldan A. (2012). Adhesion phenomena in bonded joints. Int. J. Adhes. Adhes..

[93] Allen K. (1992). Silanes as the interphase in adhesive bonds. J. Adhes. Sci. Technol..

[94] Pujol J.-M., Prébet C. (2003). Functional silanes: Crosslinkers for silicone elastomers. J. Adhes. Sci. Technol..

[95] Li M., Zhang L., Li X., Wang R., Wu X., Zhang D., Chen Y. (2023). Improvements of adhesion strength of water-based epoxy resin on carbon fiber reinforced polymer (CFRP) composites via building surface roughness using modified silica particles. Compos. Part A Appl. Sci. Manuf..

[96] Chen L., Jin H., Xu Z., Shan M., Tian X., Yang C., Wang Z., Cheng B. (2014). A design of gradient interphase reinforced by silanized graphene oxide and its effect on carbon fiber/epoxy interface. Mater. Chem. Phys..

[97] Hatefi A., Mohagheghi S., Kianvash A. (2013). The effect of silane layer drying temperature on epoxy coating adhesion on silane-pretreated aluminum substrate. J. Coat. Technol. Res..

[98] Bajat J., Milošev I., Jovanović Z., Mišković-Stan V. (2010). Studies on Adhesion Characteristics and Corrosion Behaviour of Vinyltriethoxysilane/Epoxy Coating Protective System on Aluminium. Appl. Surf. Sci..

[99] Hench L.L., Hench L.L. (1998). 2–Sol-Gel Kinetics. Sol-Gel Silica.

[100] Klein L.C. (1985). Sol-Gel Processing of Silicates. Annu. Rev. Mater. Sci..

[101] Pierre A.C., Alain C.P. (1998). Introduction to Sol-Gel Processing.

[102] Musgraves J.D., Hu J., Calvez L. (2019). Springer Handbook of Glass.

[103] Hamzaoui H.E., Bouazaoui M., Capoen B. (2020). Sol–gel materials for optical fibers. Sol-Gel Derived Optical and Photonic Materials.

[104] Atkins A.G. (1975). Intermittent bonding for high toughness/ high strength composites. J. Mater. Sci..

[105] Outwater J.O., Murphy M.C. (1970). The Influences of Environment and Glass Finishes on the Fracture Energy of Glass-Epoxy Joints. J. Adhes..

[106] Plueddemann E.P. (1991). Reminiscing on silane coupling agents. J. Adhes. Sci. Technol..

[107] Matisons J.G. (2012). Silanes and Siloxanes as Coupling Agents to Glass: A Perspective. Advances in Silicon Science.

[108] Angst D.L., Simmons G.W. (1991). Moisture absorption characteristics of organosiloxane self-assembled monolayers. Langmuir.

[109] Brook M.A. (2000). Brook and Others, Silicon in Organic, Organometallic, and Polymer Chemistry.

[110] Pantano C.G., Wittberg T.N. (1990). XPS analysis of silane coupling agents and silane-treated E-glass fibers. Surf. Interface Anal..

[111] Drown E., Al Moussawi H., Drzal L. (1992). Glass fiber ‘sizings’ and their role in fiber-matrix adhesion. J. Adhes. Sci. Technol..

[112] Wang D., Jones F., Denison P. (1992). TOF SIMS and XPS study of the interaction of hydrolysed γ-aminopropyltriethoxysilane with E-glass surfaces. J. Adhes. Sci. Technol..

[113] Feller J., Grohens Y. (2004). Coupling ability of silane grafted poly(propene) at glass fibers/poly(propene) interface. Compos. Part A Appl. Sci. Manuf..

[114] Cech V., Prikryl R., Balkova R., Vanek J., Grycova A. (2003). The influence of surface modifications of glass on glass fiber/polyester interphase properties. J. Adhes. Sci. Technol..

[115] Luo N., Zhong H., Yang M., Yuan X., Fan Y. (2016). Modifying glass fiber surface with grafting acrylamide by UV-grafting copolymerization for preparation of glass fiber reinforced PVDF composite membrane. J. Environ. Sci..

[116] Chen J., Zhao D., Jin X., Wang C., Wang D., Ge H. (2014). Modifying glass fibers with graphene oxide: Towards high-performance polymer composites. Compos. Sci. Technol..

[117] Turrión S.G., Olmos D., González-Benito J. (2005). Complementary characterization by fluorescence and AFM of poly-aminosiloxane glass fibers coatings. Polym. Test..

[118] Sarr M.M., Inoue H., Kosaka T. (2021). Study on the improvement of interfacial strength between glass fiber and matrix resin by grafting cellulose nanofibers. Compos. Sci. Technol..

[119] Mattson B., Aksnes E., Huse J., Tveten C., Redford K., Stori A. (1996). Silane-modified polymers as interphases in glass-fibre-reinforced composites: 2. Grafting and crosslinking of interphase materials. Compos. Interfaces.

[120] Sorokin A.E., Petrova G.N. (2020). Lubricants and Coupling Agents in the Processes of the Liquid-Phase Modification of the Surface of Carbon and Glass Fiber Fillers in the Production of Structural Materials: A Review. Theor. Found. Chem. Eng..

[121] Park J.H., Hwang S.-H. (2022). Construction and Characterization of Polyolefin Elastomer Blends with Chemically Modified Hydrocarbon Resin as a Photovoltaic Module Encapsulant. Polymers.

[122] Baiamonte M., Morici E., Colletti C., Dintcheva N.T. (2022). Polar Wax as Adhesion Promoter in Polymeric Blend Films for Durable Photovoltaic Encapsulants. Materials.

[123] McMahon T.J., Jorgensen G.J. Adhesion and Thin-Film Module Reliability. Proceedings of the 2006 IEEE 4th World Conference on Photovoltaic Energy Conference.

[124] Miller D.C., Annigoni E., Ballion A., Bokria J.G., Bruckman L.S., Burns D.M., Chen X., Feng J., French R.H., Fowler S. Degradation in PV encapsulant strength of attachment: An interlaboratory study towards a climate-specific test. Proceedings of the 2016 IEEE 43rd Photovoltaic Specialists Conference (PVSC).

[125] Kalbe K., Piikov H., Kesti J., Honkakoski E., Kurnitski J., Kalamees T. (2020). Moisture dry-out from steel faced insulated sandwich panels. E3S Web Conf..

[126] King A. (2018). Flash into View. Build. Connect..

[127] Watts A. (2011). Silicone-Sealed Glazing and Rooflights. Modern Construction Envelopes.

[128] Pochivalov K.V., Shilov A.N., Lebedeva T.N., Ilyasova A.N., Golovanov R.Y., Basko A.V., Kudryavtsev Y.V. (2020). Development of vibration damping materials based on butyl rubber: A study of the phase equilibrium, rheological, and dynamic properties of compositions. J. Appl. Polym. Sci..

[129] Li Z., Crocker M.J. (2005). A Review on Vibration Damping in Sandwich Composite Structures. Int. J. Acoust. Vib..

[130] Lu J., Wang L. (2004). Production and application of high strength steel sheet for automobile. Automob. Techno Mat.

[131] Roth R., Clark J., Kelkar A. (2001). Automobile bodies: Can aluminum be an economical alternative to steel?. JOM.

[132] Boon J.E., Isaacs J.A., Gupta S.M. (2000). Economic Impact of Aluminum-Intensive Vehicles on the U.S. Automotive Recycling Infrastructure. J. Ind. Ecol..

[133] Wang F., Xu J., Luo H., Wang J., Wang Q. (2009). A New Organofunctional Ethoxysilane Self-Assembly Monolayer for Promoting Adhesion of Rubber to Aluminum. Molecules.

[134] Plueddemann E.P. (1970). Adhesion Through Silane Coupling Agents. J. Adhes..

[135] Thiedmanu W., Tolan F.C., Pearce P.J., Morris C.E.M. (1987). Silane Coupling Agents as Adhesion Promoters for Aerospace Structural Film Adhesives. J. Adhes..

[136] Crook R.A., Sinclair J.W., Poulter L.W., Schulte K.J. (1998). An Environmentally-Friendly Process for Bonding Aluminum Using Aqueous Metasilicate Sol-Gel and Silane Adhesion Promoters. J. Adhes..

[137] Chen H., Wang J., Huo Q. (2007). Self-assembled monolayer of 3-aminopropyltrimethoxysilane for improved adhesion between aluminum alloy substrate and polyurethane coating. Thin Solid Films.

[138] Maege I., Jaehne E., Henke A., Adler H.-J.P., Bram C., Jung C., Stratmann M. (1998). Self-assembling adhesion promoters for corrosion resistant metal polymer interfaces. Prog. Org. Coat..

[139] Li X., Wang P., Long S., Huang Y., Li H., Hu C. (2019). Interfacial adhesion and water resistance of stainless steel–polyolefin improved by functionalized silane. Polym. Eng. Sci..

[140] Lee S.R., Bae K.M., Baek J.J., Kang M.C., Lee T.I. (2021). Adhesion enhancement between aluminum and butyl rubber by (3-mercaptopropyl) trimethoxy silane for vibration damping plate. J. Adhes. Sci. Technol..

[141] Sang J., Aisawa S., Miura K., Hirahara H., Jan O., Jozef P., Pavol M. (2017). Adhesion of carbon steel and natural rubber by functionalized silane coupling agents. Int. J. Adhes. Adhes..

[142] Nützel O., Saul R. (2015). Long-term corrosion protection for bridge cables with butyl rubber tapes using the ATIS Cableskin^®^ system. Steel Constr..

[143] Saul R., Nützel O. (2012). Wrapping with Butyl Rubber Tapes—An Innovative Corrosion Protection for Bridge Cables. Struct. Eng. Int..

[144] Rajkiewicz M., Slaczka M., Czakaj J. (2014). Adhesive Properties of The Butyl Rubber Compounds. Adv. Sustain. Pet. Eng. Sci..

[145] Rajkiewicz M., Ślączka M., Czakaj J. (2014). Part I—The Butyl Rubber Compounds. Adhesive Properties.

[146] Thomas S., Dechant D.A. (2019). 2-Layer Polyethylene Extruded Factory-Applied Pipe Corrosion Coating. Pipelines 2019.

[147] Okyere M.S. (2019). Corrosion Protection for the Oil and Gas Industry: Pipelines, Subsea Equipment, and Structures.

[148] Papavinasam S., Attard M., Revie R.W. (2008). Evolution of External Pipeline Coatings for Corrosion Protection—A Review. Corros. Rev..

[149] Perova M., Galimzyanova R., Khakimullin Y., Vol’Fson S. (2011). Influence of the Molecular Weight of Oligoisobutylenes on the Properties of Uncured Sealants. Int. Polym. Sci. Technol..

[150] Akhmedgoraeva A.R., Stytsenkov A.A., Galimzyanova R.Y., Khakimullin Y.N. (2019). The Influence of Thickness and Reinforcement on the Properties of Sealing Tapes of Incongealable Type on the Basis of Butyl Rubber Depending on the Nature of the Glued Substrate. Polym. Sci. Ser. D.

[151] Kempe M.D., Dameron A.A., Reese M.O. (2013). Evaluation of moisture ingress from the perimeter of photovoltaic modules. Prog. Photovolt. Res. Appl..

[152] Tian Y., Guo L. (2020). Adhesion performance of UHMWPE fiber treated with polyethylene wax grafted methyl methacrylate alone or in conjunction with silane coupling agent. J. Adhes. Sci. Technol..

[153] Guo Y., Li Y., Wang S., Liu Z.-X., Cai B., Wang P.-C. (2019). Effect of silane treatment on adhesion of adhesive-bonded carbon fiber reinforced nylon 6 composite. Int. J. Adhes. Adhes..

[154] Lee J.W., Heo J.H., Lee B., Cho H.H., Kim T., Lee J.H. (2019). Enhancement in the adhesion properties of polycarbonate surfaces through chemical functionalization with organosilicon coupling agents. J. Mater. Sci. Mater. Electron..

